# Chloramphenicol Derivatives as Antibacterial and Anticancer Agents: Historic Problems and Current Solutions

**DOI:** 10.3390/antibiotics5020020

**Published:** 2016-06-03

**Authors:** George P. Dinos, Constantinos M. Athanassopoulos, Dionissia A. Missiri, Panagiota C. Giannopoulou, Ioannis A. Vlachogiannis, Georgios E. Papadopoulos, Dionissios Papaioannou, Dimitrios L. Kalpaxis

**Affiliations:** 1 Department of Biochemistry, School of Medicine, University of Patras, GR-26504 Patras, Greece; dinosg@upatras.gr (G.P.D.); bio3320@upnet.gr (P.C.G.); vlacho.giannis92@gmail.com (I.A.V.); 2 Laboratory of Synthetic Organic Chemistry, Department of Chemistry, University of Patras, GR-26504 Patras, Greece; dionysia.misiri@gmail.com (D.A.M.); dapapaio@upatras.gr (D.P.); 3 Department of Biochemistry and Biotechnology, University of Thessaly, Ploutonos 26, GR-41221 Larissa, Greece; geopap@uth.gr

**Keywords:** chloramphenicol, antibiotics, antibiotic resistance, side effects, anticancer agents, chemical synthesis, translation, ribosome, peptidyl transferase, puromycin reaction

## Abstract

Chloramphenicol (CAM) is the D-*threo* isomer of a small molecule, consisting of a *p*-nitrobenzene ring connected to a dichloroacetyl tail through a 2-amino-1,3-propanediol moiety. CAM displays a broad-spectrum bacteriostatic activity by specifically inhibiting the bacterial protein synthesis. In certain but important cases, it also exhibits bactericidal activity, namely against the three most common causes of meningitis, *Haemophilus influenzae*, *Streptococcus pneumoniae* and *Neisseria meningitidis*. Resistance to CAM has been frequently reported and ascribed to a variety of mechanisms. However, the most important concerns that limit its clinical utility relate to side effects such as neurotoxicity and hematologic disorders. In this review, we present previous and current efforts to synthesize CAM derivatives with improved pharmacological properties. In addition, we highlight potentially broader roles of these derivatives in investigating the plasticity of the ribosomal catalytic center, the main target of CAM.

## 1. Introduction

Sixty decades of biochemical studies and more recent crystallographic studies, have revealed the molecular basis by which chloramphenicol (CAM) specifically inhibits bacterial protein synthesis. CAM is an old broad-spectrum antibiotic but its medical and veterinary applications as antibacterial are full of pros and cons. Besides resistance to CAM frequently reported, the major disinclined concerns for the clinical use of CAM relate to the side effects in long antibiotic courses causing neurotoxicity and hematologic disorders. This has prompted many investigators to attempt the synthesis of new CAM derivatives with improved pharmaceutical properties. In this review, we discuss how the information gained from these efforts has advanced our knowledge on the mode of action of CAM and how these CAM derivatives have contributed to compacting resistant bacteria and addressing side effects of the drug.

## 2. Mode of Action of CAM

**Figure 1:**
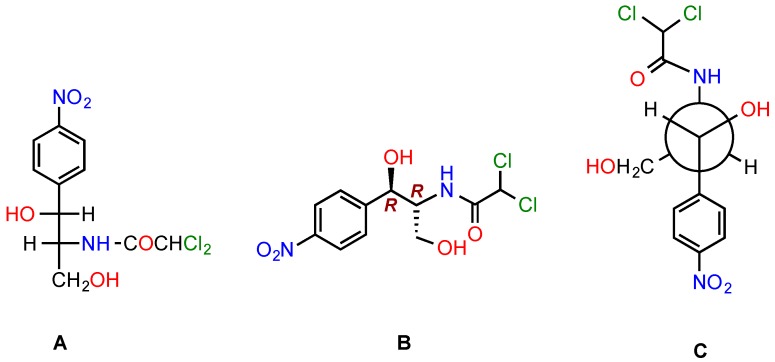
Alternative structures for CAM. (**Α**) Fischer projection (D-*threo* isomer); (**Β**) Skeletal formula showing the configuration of the two stereogenic centers; (**C**) Newman projection.

Upon CAM discovery [1], it was recognized that the D-*threo* isomer (Figure 1) inhibits bacterial protein synthesis. The other stereoisomers of CAM, not occurring in nature, were found inactive [2].

**Figure 2:**
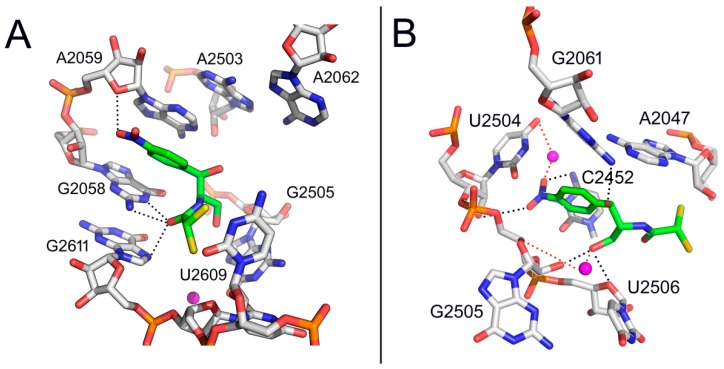
Binding positions of CAM (in green) in the (**A**) *Haloarcula marismortui* and (**B**) *Deinococcus radiodurans* ribosome, as detected by crystallography (PDB ID code 1NJI and 1K01, respectively).

Two binding sites in ribosomes were identified by early equilibrium studies [3], CAM1 and CAM2, with affinity constants equal to 2 μΜ and 200 μΜ, respectively. Recently, cross-linking of CAM to ribosomes isolated from the bacterium *Escherichia coli* and the archaeal *Halobacterium halobium* localized CAM2 at the entrance to the ribosomal peptide exit tunnel [4]. A similar binding site was observed by crystallography in the *Haloarcula marismortui* ribosome, overlapping the macrolide binding site [5] (Figure 2A). Nevertheless, high concentrations of CAM were used by both studies to cross-link or bind the drug at the exit-tunnel, a fact that limited the functional significance of CAM2 binding site.

Quite recently we revealed a second kinetically cryptic binding site for CAM at the nucleotide G2611 of the 50S ribosomal subunit of *E. coli*, using much lower concentrations (<60 μΜ) of a CAM homodimer, than those utilized previously [6]. A plausible explanation for this raised affinity is that binding of the dimer to the high affinity site (CAM1) facilitates targeting of a cryptic, low affinity site (CAM2) via the second edge of the homodimer. Consistently, erythromycin, a macrolide that binds at the entrance of the exit tunnel, in turn inhibits binding of CAM [7], while CAM enhances the premature release of short oligopeptidyl-tRNAs, reminiscent of macrolide antibiotics [8]. Nevertheless, due to its low affinity for CAM, the CAM2 binding site seems unlikely to play a central role in inhibition of protein synthesis, an hypothesis also strengthened by the fact that most of the resistance mutations or modifications cluster around the CAM1 binding site [9,10,11,12].

In contrast to the CAM2 binding site, CAM1 seems to be strongly relevant for inhibition. Numerous biochemical and structural studies, like competition studies between CAM and small tRNA fragments for the A-site of the peptidyl transferase (PTase) center [13], inhibition of the puromycin reaction by CAM [14,15,16,17], and crystallographic analyses of CAM bound to 50S subunits from bacteria [18,19,20] demonstrated that the CAM1 binding site lies within the ribosomal A-site (Figure 2B).

**Figure 3:**
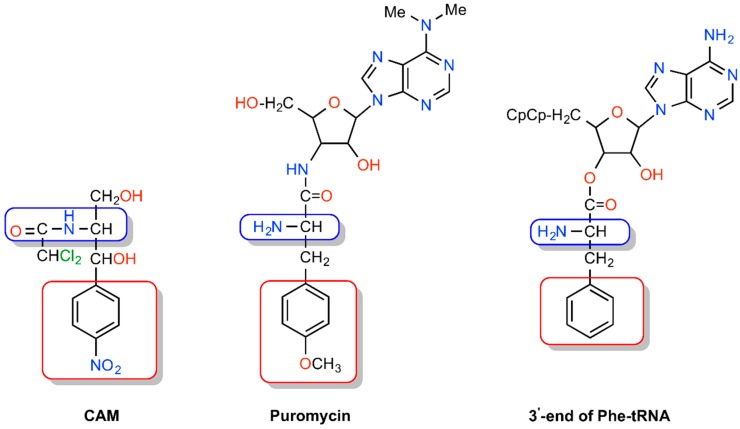
Structures of CAM, puromycin and Phe-tRNA^Phe^ in an iso-structural orientation.

CAM has been considered as iso-structural to both puromycin and the 3′-end of aminoacyl-tRNA (Figure 3) [21]. The initial observation in favor of this consideration was undertaken in a computational study that related CAM to the peptide moiety of peptidyl-tRNA bound to the P-site and the corresponding transition state of peptide bond formation with aminoacyl-tRNA [22].

**Figure 4:**
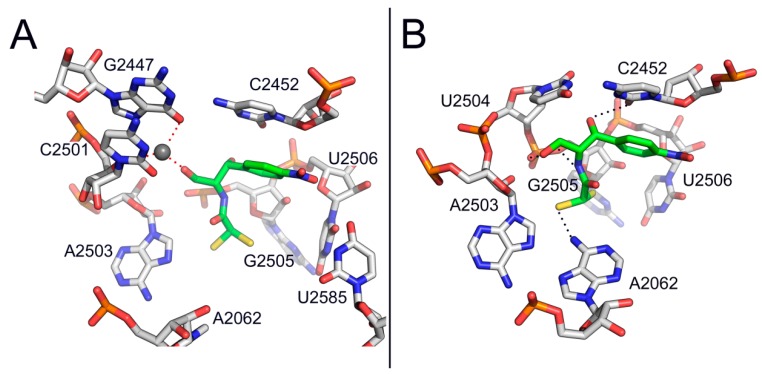
Binding positions of CAM (in green) in the (**A**) *Thermus thermophilus* and (**B**) *Escherichia coli* ribosome, as detected by crystallography (PDB ID code 4V7W and 3OFC, respectively).

Nevertheless, this proposal ignored that CAM is essentially an A-site inhibitor. A few years later, Bhuta *et al.* [23] oriented the *p*-nitrophenyl group of CAM and the methylated-tyrosyl moiety of puromycin to the hydrophobic crevice of the A-site, so that the CO-NH sequence of the amido bonds in puromycin and CAM were in the opposite direction. Thus, CAM and puromycin were regarded as *retro-inverso* analogs, which is at variance with a crystallographic model of CAM in complex with *Deinococcus radiodurans* 50S subunit (Figure 2B) [18] but in line with current crystallographic evidence from CAM-50S ribosomal subunit (Figure 4) complexes from *Thermus thermophilus* and *E. coli* [19,20]. The concept of *retro-inverso* orientation of CAM in relation to the substrate of the A-site is particularly important if we take into consideration that *retro-inverso* analogs of peptides exhibit high biological activity [24].

In the course of kinetic studies, early evidence suggested that CAM behaves as a simple competitive inhibitor of peptide bond formation, a view compatible with the structural similarity of CAM with puromycin, a pseudo-substrate of A-site of PTase and the 3′-end of aminoacyl-tRNA (Figure 3) [25,26]. However, besides competitive kinetics, there is also evidence for noncompetitive [15] or mixed-noncompetitive type of inhibition mechanism [14,16,27], depending on the buffer ionic conditions used, the inhibitor concentration, the pre-incubation effect and the nature of the substrates. Evidence for the dependence of CAM inhibition kinetics on mono- and divalent cations was provided by high-resolution structures of CAM-ribosome complexes, indicating the involvement of cations mediating coordination bonding between the drug and ribosomal residues [18,20]. The inhibitor concentration effect and the significance of ribosome pre-incubation with the drug before adding the substrate were understood when it was realized that CAM behaves as a slow-binding competitive inhibitor of peptide bond formation, acting via a two-sequential reaction; CAM (I) reacts rapidly with an initiator ribosomal complex (C) to form the encounter complex CI that is then isomerized slowly to a more tight complex C*I [17]. Consistently, as observed in some studies, noncompetitive or mixed-noncompetitive modes of action could be explained by the slow-onset inhibition theory [28] according to which the inhibition pattern under “pre-incubation” conditions can be misinterpreted as indicating mixed, noncompetitive inhibition. Regarding the effects of the nature of the substrate, it was observed that tRNAs bearing bulky amino acids, like Phe-tRNA^Phe^ are less prone to inhibition by CAM than tRNAs carrying smaller or charged amino acids [8,29]. The nature of the P-site bound substrate can also influence CAM inhibitory activity, an effect consistent with the crystallographic observation that the *p*-nitro moiety of CAM is in close proximity to the aminoacyl moiety of P-site bound tRNA [19,20].

Beyond the inhibition of peptide bond formation, CAM can perturb additional ribosomal functions, such as termination [30], translational accuracy [31] and biogenesis of 50S ribosomal subunits [32]. With regards to the latter study, more recent observations suggested that ribosomal assembly defects are due to a specific inhibition of the biosynthesis of ribosomal proteins by CAM [33].

## 3. Bacterial Survival Strategies to Combat CAM Activity

The misuse of antibiotics in clinical practice and the widespread use in agriculture has seriously contributed to the rise in global resistance to antibiotics. Nevertheless, the microbial world has had *per se* the molecular tools to drive resistance; the antibiotic resistome pre-dates the modern antibiotic era by millions of years. Therefore, development of resistance cannot be completely eradicated and is only a matter of when. The antibiotic-resistance crisis is further aggravated by the paucity of new antibiotics in the pipeline [34].

Resistance or decreased sensitivity to CAM has been frequently observed and is mediated by numerous mechanisms. Target mutations or alterations are a usual mechanism of bacterial resistance to CAM. At least eight mutations in the V domain of 23S rRNA and one methyl-modification of A2503 confer high CAM resistance in bacteria [9,10,11,12], especially when occurring in most of rRNA operons (7 in *E. coli*). More crucial are mutations and/or deletions of ribosomal proteins, which are usually encoded by single gene copies. However, mutations in ribosomal proteins causing CAM-resistance have been identified only in a few cases so far [35,36]. Because interactions of CAM with ribosomal proteins have not been detected by crystallographic analyses [18,20], such resistance is suspected to arise indirectly, probably by induced conformational changes in rRNA. It has been found that combination of both ribosomal protein and rRNA mutations lead to increased resistance; e.g. G-to-A nucleotide change at position 2073 (2058, *E. coli* numbering) in all three copies of the 23S rRNA gene in combination with G14D modification in ribosomal protein L4 confers CAM resistance in *Campylobacter jejuni* [36]. Nevertheless, such mutations are often accompanied by fitness cost and consequently are not extensively spread.

The most frequently encountered mechanism of bacterial resistance to CAM is the enzymatic inactivation of the drug warhead by different types of enzymes, extensively reviewed by Schwarz *et al.* [37]. Although O-phosporylation [38,39,40], hydrolytic degradation to *p*-nitrophenylserinol [41,42], and nitroreductation [43] have been reported previously, the major enzymatic mechanism that inactivates CAM is acetylation via several types of CAM acetyltransferases (CATs). All CAM acetyltransferases share similar molecular strategies in acetylating CAM. The O-acetyl derivatives of CAM fail to act as antibiotics, because they do not bind to bacterial ribosomes. 

Beyond the above mechanisms, there are also publications on other mechanisms of CAM resistance, such as permeability barriers and efflux systems. CAM gains access to the periplasm through pore-forming porins [44]. Permeation of the outer membrane by antibiotics through porins depends on the molecular dimensions of the drugs [45]. Therefore, perturbations of the outer membrane or derivatization of CAM may reduce its capacity for internalization into the cell. On the other hand, bacteria defend themselves against antibiotics by pumping out the drugs. Export of CAM from the bacterial cell can be mediated either by specific and/or multi-drug efflux pumps, the former mediating higher levels of resistance compared to those of nonspecifically driving out the drug [37]. Genes associated with CAM specific efflux pumps have been identified and characterized in a wide variety of bacteria. Eight different groups of specific exporters, reviewed by Schwarz *et al.* in 2004 [37], are constantly enriched by new data [46,47]. CAM is a substrate of most multi-drug efflux pumps, namely the major facilator superfamily (MPS) [48], the small multi-drug resistance (SMR) family [49], the resistance nodulation and cell division (RND) family [50], the ATP-binding cassette (ABC) family [51] and the multidrug and toxin extrusion (MATE) family [52]. Worth noting, CAM may induce expression of efflux pumps, such as MexXY in *Pseudomonas aeruginosa* that belongs to RND family [53] or enhance the efflux of other ligands by AcrB pump in *E. coli* [54]. Such effects, not beginning nor ending with these special examples, complicate the use of CAM for the treatment of bacterial infections. 

## 4. Side Effects of CAM

Soon after its discovery and subsequent synthesis, CAM became very popular due to its antimicrobial effectiveness and low cost. However, in the following years when CAM was issued for general clinical use, it was realized that CAM can cause hematological disorders like bone marrow depression and aplastic anemia [55]. While an irreversible and fatal aplastic anemia appeared to be a rare complication, probably caused by nitrobenzene metabolites of CAM that act on DNA [56,57,58], mild and reversible bone marrow suppression was found to occur quite frequently and was ascribed to a mitochondrial protein synthesis inhibition, particularly in cases in which underlying mitochondrial mutations favor CAM binding to mitochondrial ribosome (mitoribosome) [59,60]. Despite mitochondria may have originated from bacteria (endosymbiosis hypothesis), the mitochondrial ribosomal machine is substantially different from that found in bacterial and human cells [61]. Nevertheless, the catalytic core of PTase and decoding center architecture is conserved in mitoribosome and bacterial ribosome. This explains why CAM is capable of inhibiting mitochondrial protein synthesis. Further studies in mice indicated that CAM inhibits protein synthesis in mitoribosomes of the marrow progenitor cells at the differentiation rather than the replication stage [62]. Due to its lipoidal nature (MLogP = 1.23), low number of OH and NH H-bond donors (=3) and low number of N and OH-bond acceptors (=7), CAM penetrates well in all tissues, including many privileged sites such as the bone marrow and the brain [63]. It remains relatively unbound to proteins and its small molecular size (MW = 323.14) facilitates crossing of the blood-brain barrier (BBB) [64]. Penetration of BBB was further improved when optimized palm kernel oil esters nanoemulsion-loaded with chloramphenicol were used to cross BBM [65]. These properties made the CAM-loaded nanoemulsions a first-choice therapeutic for meningitis treatment. Although less available in brain and cerebrospinal fluid (CSF) relative to plasma, CAM can achieve high levels in brain and CSF after prolonged use and high cumulative dose that may cause drug-related mitochondrial neuropathies [66]. CAM treatment over a long period may also cause irreversible panmyelopathy which is lethal and may be associated with genetic defects. Dermatological studies disclosed lymphocyte sensitization by chloramphenicol and azidamphenicol in allergic contact dermatitis [67], while experiments in mice splenocytes revealed immunosuppressive activity of a CAM analog, florfenicol, on the immune response to stimulators [68]. In neonates and infants lacking glucuronidation reactions (phase II detoxification) and sufficient renal capacity for excreting unconjugated drugs, accumulated toxic CAM metabolites can cause Gray syndrome, a disease manifested by hypotension, cyanosis, hypothermia, cardiovascular collapse, ashen gray color of the skin and vomiting [69]. There is also evidence that CAM causes mitochondrial stress that in turn promotes matrix metalloproteinase-13-associated cancer cell invasion [70]. Due to the above harmful effects, the clinical use of CAM is currently limited in developed countries to infections not responding to other antibiotics. Nevertheless, a recent study suggests that the inhibitory effects of antibiotics on the host mitochondrial protein synthesis and the subsequent negative consequences on mitochondrial biogenesis would allow us to eradicate cancer stem cells, across multiple tumor types, by repurposing antibiotics for anti-cancer therapy [71]. This suggestion was based on the hypothesis that cancer stem cells are strongly anabolic and they may require robust mitochondrial biogenesis and activity for survival and proliferative expansion. Indeed, treatment of MCF7 cells with CAM caused inhibition of tumor-sphere formation with an IC_50_ of approximately 200 μΜ. Consistently, previous *in vitro* studies have indicated that CAM alone, or in combination with other anticancer drugs can cause inhibition of growth in several cancer lines including leukemic cell lines [6,72,73]. Taking into account the critical role of CAM in induced leukemogenesis [74], it is clear that additional cellular studies using different solid tumor cell lines and graded doses of CAM are needed to explore the potentially beneficial role of CAM in cancer therapy.

To define which regions of the parent molecule are essential for antibacterial activity or strongly associated with toxicity, numerous CAM analogs have been synthesized and biologically evaluated. For the sake of brevity, we present below only the most important of them, particularly those investigated as drugs.

## 5. Modifications of CAM to Obtain Antibacterials with Improved Properties

### 5.1. Modifications of the p-Nitrophenyl Moiety

**Figure 5:**
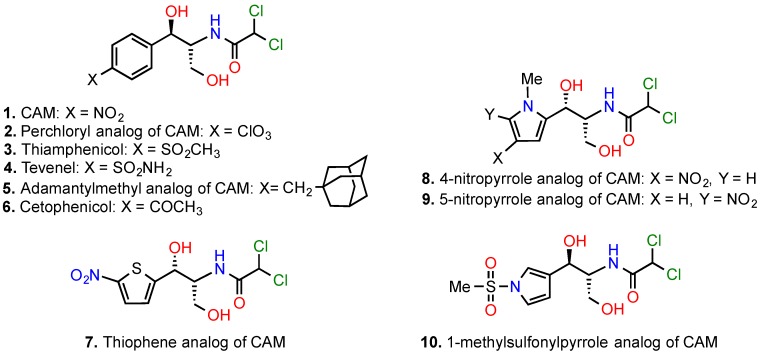
Analogs of CAM derived through modification or replacement of the *p*-nitrobenzene moiety.

Soon after recognizing that the specific metabolism of the nitrobenzene ring system causes the appearance of partial or total reduction products in the human body, like nitroso-compounds and hydroxylamine-derivatives, capable of causing DNA damage and irreversible aplastic anemia, the interests of the scientific community orientated to the development of CAM derivatives with changes in the p-nitrobenzene moiety, by substituting the nitro group with a number of other electron withdrawing groups (Figure 5; compounds **2**–**6**) or by replacement of the whole aromatic system (Figure 5; compounds **7**–**10**).

Most of them exhibited gentler toxicity, without severe loss of pharmaceutical potency [2,57]. A very potent member in this class of derivatives was the perchloryl analog of CAM (compound **2**), with a twofold larger activity than that of CAM. However, this derivative was never introduced in therapy, due to its explosive properties [75]. The only member that received clinical applications was thiamphenicol (compound **3**). This compound has never been associated with aplastic anaemia, despite its extensive use in human [76]. Nevertheless, its weaker antibacterial activity, compared to CAM, and reversible toxicity on bone marrow and kidneys have currently shifted its use primarily for veterinary. Tevenel (compound **4**), although stronger inhibitor of peptide bond formation *in vitro* than thiamphenicol [16], was also found to be a potent inhibitor of mitochondrial protein synthesis and was never used on humans.

An effort by Mullen and Georgief to replace the nitro group of CAM with a bulky nonpolar substituent, namely the adamantylmethyl group (compound **5**), was detrimental for antibacterial activity [77]. This replacement decreases the hydrophilicity of the molecule, but increases the molecular size that negatively correlates with bacterium penetration [78]. Today, it is easy to explain this failure for an additional reason; recent crystallographic studies revealed that a bulky substituent of the benzene ring at the *p*-site could hamper stacking of the aromatic ring on C2452 of 23S rRNA [19,20].

Synthesis of pyrrole or thiophene analogs of CAM has been also reported [57,79]. Some of them are illustrated in Figure 5 (compounds **8**–**10**). Compound **10**, in which the *p*-nitrophenyl moiety of CAM was replaced by 1-methylsulfonylpyrrole was found to exhibit *in vitro* activity close to that of thiamphenicol, suggesting that the *p*-nitrobenzene moiety of CAM is susceptible of replacement by alternative aromatic systems. Although compound **10** was less toxic than CAM, it has never had therapeutic applications.

### 5.2. Modifications of the 2-Amino-1,3-propanediol Moiety

**Figure 6:**
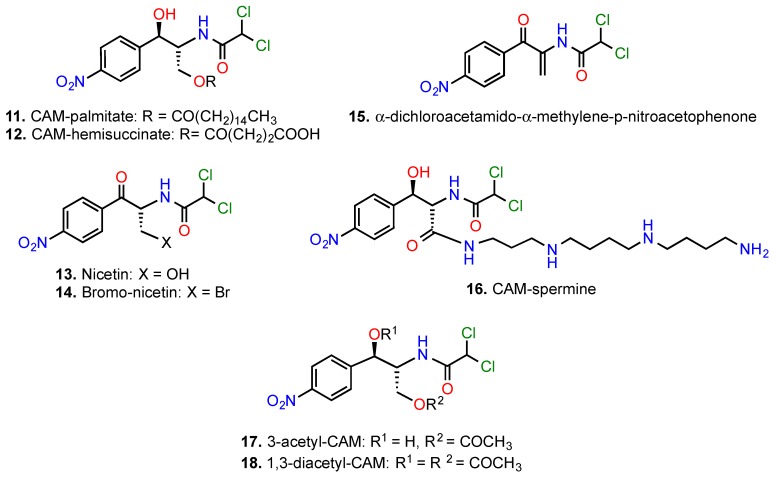
Derivatives of CAM modified at the 2-amino-1,3-propanediol moiety.

Early efforts to improve the pharmacokinetic properties of CAM indicated that substitutions in the 2-amino-1, 3-propanediol moiety are associated with severe loss of biological activity [2]. Exceptionally, three examples were deviated from this rule. First, various hydrolysable esters of CAM were prepared (Figure 6), useful for oral administration. They were found to be easily hydrolyzed into free CAM in the gastrointestinal tract (CAM-palmitate, compound **11**) or in plasma, liver, lungs and kidneys (CAM-hemisuccinate, compound **12**) [80]. Second, transformation of the secondary OH group into a keto group with consequent elimination of stereoisomerism, led to the synthesis of nicetin (compound **13**), an efficacious drug for the treatment of fungous infections of the skin [81]. Two relative compounds, additionally modified at the OH group in position 3, that is compounds **14** and **15**, were found to be many times more active than CAM in combating resistant strains overexpressing acetyltransferase activity [82,83]. The high efficacy of compounds **14** and **15** may be attributed to the modifications at positions 1 and 3 of CAM that prevent enzymatic inactivation of the drug by acetyl- and phospho-transferases.

Bulky substitutions at positions 1 and 3 lead to an opposite effect. For instance, we recently prepared a CAM-polyamine conjugate by introducing a spermine molecule into position 3 of the 1,3-propanediol backbone of CAM, following oxidation of the primary hydroxyl group (compound **16**) [73,84]. We anticipated that the conjugated polyamine portion could enhance the binding of CAM to the ribosome, by mimicking the function of an analogous cationic center found by crystallography to coordinate the interaction between the OH group at position 3 of CAM and nucleotide U2506 in *D. radiodurans* [18]. Unfortunately, the activity of compound **16** was found lower than expected. Our failure can be explained by a more recent crystallographic study conducted in *E. coli* ribosome [19]; the new orientation of CAM is rotated by 180°, thus directing the OH group in position 3 of CAM away from U2506 (Figure 4B). Noteworthy, CAM efficacy is decreased by mono-acetylation (compound **17**) and nearly disappeared with di-acetylation (compound **18**) [85].

### 5.3. Modifications at Both the p-Nitrophenyl and the 2-Amino-1,3-propanediol Moieties

**Figure 7:**
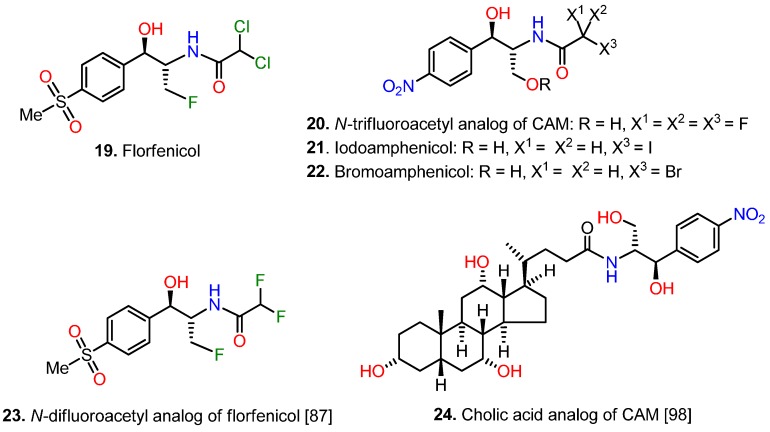
Florfenicol and derivatives of CAM modified at the dichloroacetyl moiety.

The necessity for avoiding both toxic effects and resistance to CAM led to the synthesis of florfenicol (Figure 7, compound **19**). Florfenicol was prepared from thiamphenicol by replacing the OH group in position 3 by a fluorine atom. Florfenicol was found to be a broad spectrum antibiotic *in vitro*, with activity similar or stronger than those of CAM and thiamphenicol [83,86,87,88]. However, the most interesting pharmaceutical features of florfenicol were first, its ability to combat CAM- and thiamphenicol-resistant strains bearing plasmids that possess *cat* gene and second, a more favorable toxicity profile than CAM, due to the lack of an aromatic nitro group [83,88]. In fact, florfenicol cannot deal with strains bearing resistance genes, like *floR*, encoding protein components of drug exporters [46,47,89,90,91,92], or mutations/modifications of the target [93]. Due to its favorable pharmacokinetics, florfenicol like CAM penetrates well into CSF of calves, appearing an availability of 46% relative to plasma. Therefore it has been introduced to treat meningitis in cattle and pigs [94]. It is expected that florfenicol use will extend to human medicine in the near future.

### 5.4. Modifications at the Dichloroacetyl Moiety

The dichloroacetyl moiety of CAM is essential for antibacterial potency [2]. However, numerous studies have been published in which CAM is modified by substituting this portion of the molecule. Early studies investigated the incidence of replacing the dichloroacetyl moiety by a mono- or polysubstituted with F, Br, or I acetyl group (Figure 7, compounds **20**–**23**) [2,86,87,95,96,97]. All of them were found less active than CAM. Interestingly, compounds **21** and **22** with a haloacyl tail are chemically reactive and have been employed as affinity probes of the CAM binding site in ribosomes [2]. Compound **22** was found to be an irreversible inhibitor of CAM acetyltranferase and used as affinity probe of the catalytic site of this enzyme [97]. Compound **23** is a fluorinated analog of florfenicol with less activity than CAM or florfenicol [86,87].

Prompted by the structural similarity of CAM to the 3′-terminus of aminoacyl- or peptidyl-tRNA, many investigators synthesized various (amino)acyl- and peptidyl-analogs of CAM and evaluated the biological activity of the conjugates [23,98,99,100,101,102,103,104,105,106]. Harza *et al.* synthesized a series of amides derived from CAM base and cholic acid or deoxycholic acid [98]; one of them, compound **24**, is illustrated in Figure 7. It was expected that these derivatives of CAM could easily penetrate the bacterial membranes and inhibit cell growth. Although active against various Gram-positive bacteria, their potency was much less than that of CAM.

**Figure 8:**
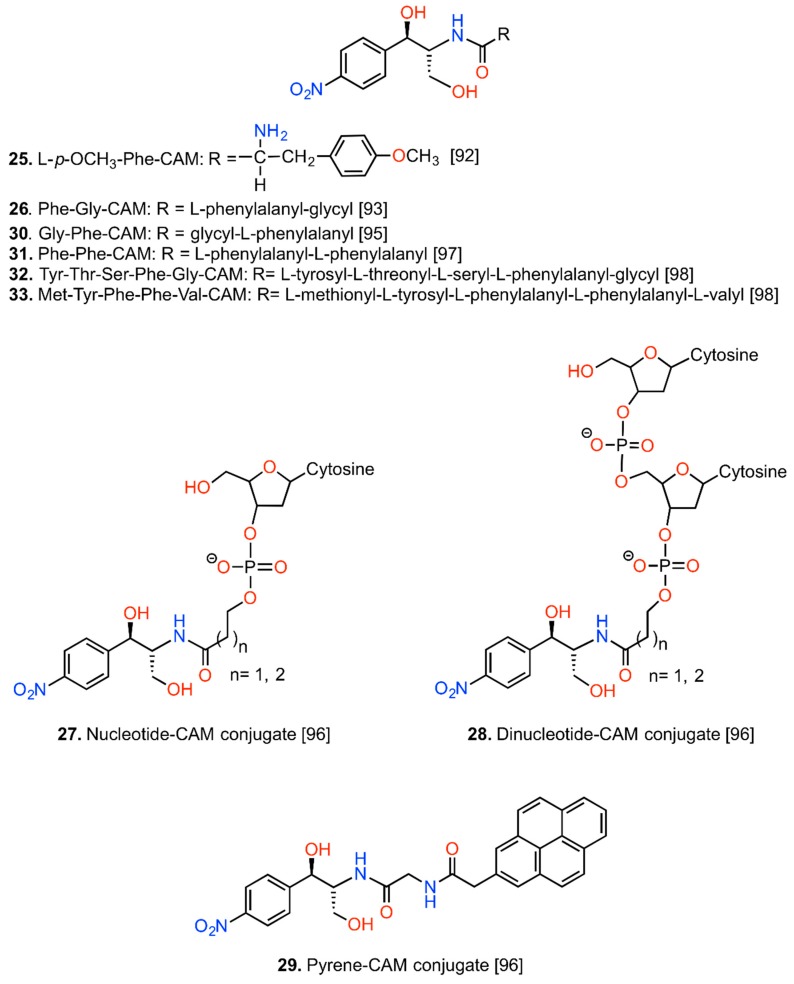
Conjugates of CAM with amino acids, peptides, nucleotides and pyrene.

The synthesis of aminoacyl and peptidyl conjugates of CAM was originally started in order to enrich the construct with additional binding sites, through the introduction of amino and carboxyl functions. However, none of them was found to have superior activity than CAM, particularly *in vivo*. Some of the most active conjugates are illustrated in Figure 8.

Correlation of the *in vitro* and *in vivo* activities led to the conclusion that steric properties of the acyl moiety significantly influence the antibacterial potency of the constructs [2]. Nevertheless, these efforts had a great contribution in understanding the mode of action of CAM at a time when information from crystallographic works was missing. More specifically, the group of Vince using a set of analogs of puromycin and CAM proposed that the amino-acylated tail of CAM simulates the aminoacyl-tail of tRNA placed at the A-site in a parallel orientation, a hypothesis extended to the suggestion that the dichloroacetyl tail of CAM points away from the exit tunnel, towards the CCA end of an incoming aminoacyl-tRNA [100,101]. Based on this proposal and the *D. radiodurans* model [18], Johansson *et al.* designed a set of nucleotide CAM conjugates that met little success (Figure 8, compounds **27** and **28**) [104]. Another CAM analog synthesized in the framework of this study, CAM-pyrene conjugate (compound **29**), was found to protect U2506 from CMCT modification. As U2506 is known to be a footprint signal of CAM binding, the authors supposed that compound **29** may bind to the drug site. However, other expected interactions between compound **29** and the ribosome were not clearly demonstrated, nor the effect on the growth of bacterial cells was further investigated. In contrast, Bhuta *et al.* using another set of aminoacyl-CAM analogs proposed that CAM binds to the ribosome in a *retro-inverso* orientation to the aminoacyl-tRNA [23]. This hypothesis that is compatible with recent crystallographic studies [19,20] was adopted by other groups to explain in a molecular basis, the activity of aminoacyl and peptidyl constructs of CAM [103,105,106].

Some of the pentapeptidyl conjugates of CAM synthesized by Mamos *et al.* showed strong affinity for the binding site of CAM (e.g. compound **32**). More interestingly, compound **33** was found to orientate its peptidyl-chain into the ribosomal tunnel and establish interactions with the region surrounding nucleotide A752 in domain II of 23S rRNA [106]. Similar interactions of a series of stalling peptides with this region of the exit tunnel have been found to inactivate allosterically the A-site of PTase [107]. Recent studies have been focused on a special class of antimicrobial peptides, the proline-rich antimicrobial peptides, which are actively transported inside the bacterial cell, bind into the exit tunnel of the ribosome and influence the function of the A-site [108]. Therefore, lessons learned from both studies could promote the development of new drugs, especially those targeting Gram-negative bacteria. 

**Figure 9:**
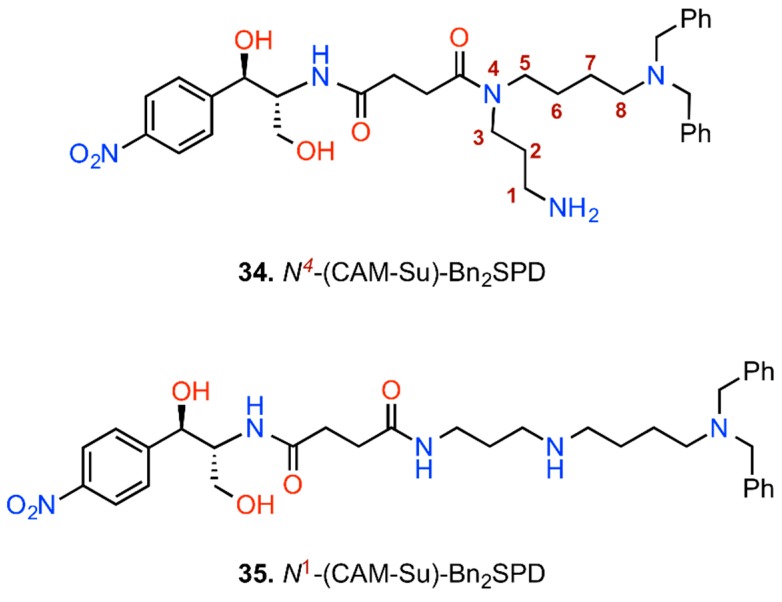
CAM-polyamine conjugates.

Due to the cellular origin of polyamines (PAs), numerous derivatives and analogs of PAs have been synthesized for medicinal purposes. An advantage of these compounds is that they are internalized into the bacterial cell by destabilizing the liposaccharide layer of the outer membrane [109] and penetrating the bacterial inner membrane through specific uptake systems [110]. The eukaryotic cells also have available specific polyamine transporters, however their molecular demands for selective delivery of PAs differ. Therefore, conjunction of CAM with PAs could facilitate the import of constructs into the cells. However, the role of PAs not merely serves as means of a “Trojan Horse”; PAs could provide the constructs with additional binding sites through their amino functions or other incorporated groups. The above information combined with the observation that PAs are implicated in the binding of CAM to bacterial ribosome [17], motivated us to design and synthesize recently a series of CAM-PA conjugates [73,84]. The most potent members of this series are shown in Figure 9 (compounds **34** and **35**). Compounds **34** and **35** were synthesized by attaching the CAM base on N^4^ and N^1^ positions of the *N*^8^,*N*^8^-dibenzylspermidine (Bn_2_SPD), respectively, via a succinate (Su) linker.

**Figure 10:**
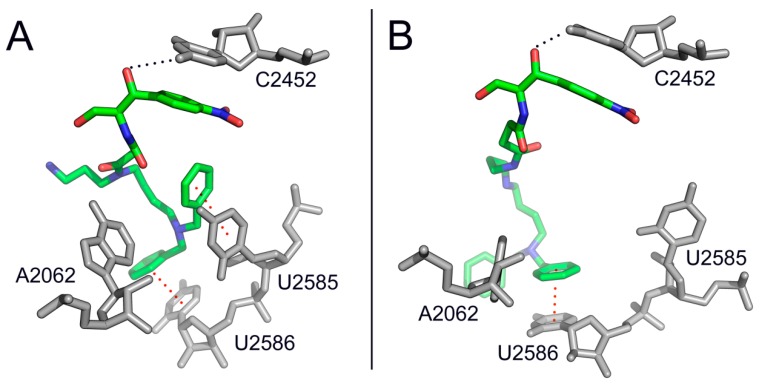
Binding positions of compounds **34** and **35** in the *Escherichia coli* ribosome, as detected by kinetic analysis, footprinting assays, and MD simulations [73]. (**A**) Compound **34** (in green) binds to the ribosomal A-site through its CAM scaffold, while the dibenzyl-PA tail makes π-stacking interactions with nucleotides U2585 and U2586 of 23S Rrna; (**B**) Compound **35** (in green) exhibits a similar pattern of interactions with the A-site but the dibenzyl-PA tail stacks only on U2586, thus behaving as a weaker inhibitor of peptide-bond formation.

Uptaken by *E. coli* cells through the SPD-preferential transport system, both compounds decreased the protein and PA content of the cells [84]. The antibacterial activity of compounds **34** and **35** was comparable or superior to that of CAM, particularly against CAM-resistance strains. Moreover, these compounds exploiting the highly active PA-transporters in cancer cells [111] and their capability to recognize the ionic surface of mitochondria, were toxic against HS-Sultan and Jurkat cells and human mesothelioma cells ZL34. Instead, their toxicity against healthy human cells was low to negligible [73]. Kinetic and footprinting analysis disclosed that the CAM-scaffold of the constructs binds to the A-site of PTase, while the PA-tail interacts with nucleotides U2585 and U2586 of 23S rRNA that interfere with the rotary motion of the 3′-terminus of aminoacyl-tRNAs (Figure 10). Current efforts by our research team to improve the lead compound **34** led to a slight strengthening of the targeting properties, but failed to improve the antibacterial potency of the construct (unpublished results), thus emphasizing the need to touch up the internalization procedure.

## 6. CAM Hybrids and Dimers

### 6.1. CAM Hybrids

Segments from two antibiotics covalently connected into one molecule and inhibiting dissimilar targets in the ribosome named hybrid antibiotics; and may show improved properties including enhanced affinity for the target, reduced potential for generating drug-resistance, elevated activity against drug-resistance strains and broader spectrum of activity.

**Figure 11:**
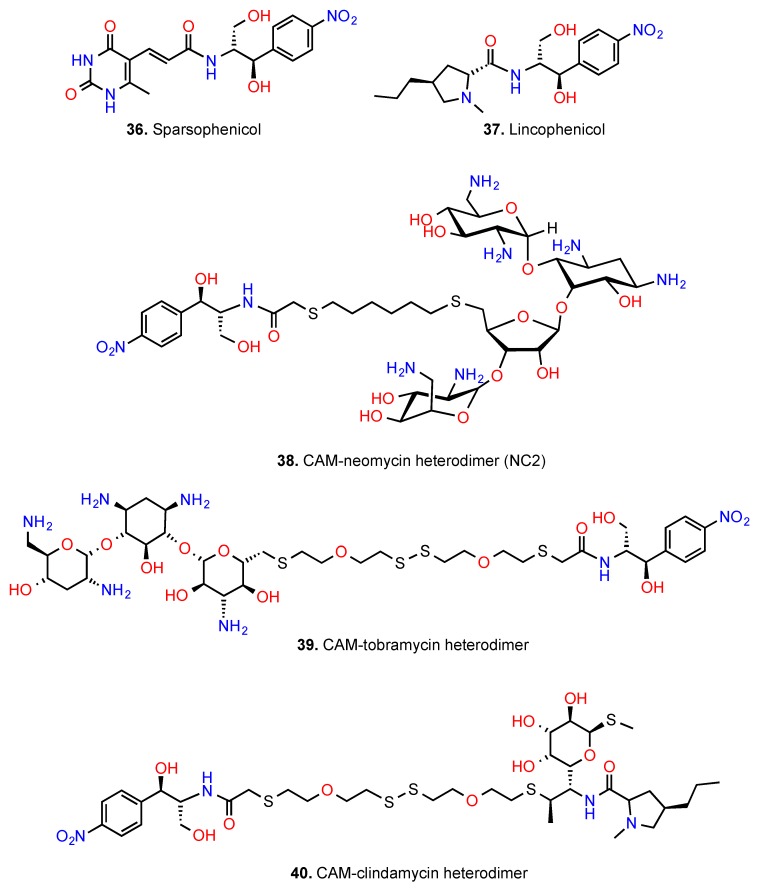
CAM hybrids and heterodimers.

Sparsophenicol (Figure 11, compound **36**) was the first CAM hybrid that was synthesized and evaluated [112]. It strongly inhibited the puromycin reaction (K_i_ = 3 μΜ) but failed to inhibit the poly(Phe) synthesis and the growth of bacterial cells, including *E. coli* strains. It was suggested that sparsophenicol is capable of inhibiting the synthesis of only a single peptide bond but is unable to penetrate the bacterial envelope, as it is less lipophilic than CAM. Sparsophenicol was devoid of *in vitro* antitumor activity when tested in inhibiting the growth of murine leukemia L1210 cells [112]. Whether the latter finding is related to the low lipophilic character of sparsophenicol or inability of this agent to target the eukaryotic cell was never explored. Another CAM hybrid combining structural features of CAM and lincomycin, named lincophenicol (compound **37**), was found to share similar activity profiles with CAM in inhibiting the puromycin reaction, poly(Phe) synthesis, and the growth of bacterial cells [113]. Both compounds **36** and **37** were not further investigated as drugs but rather used as tools for the verification of the *retro-inverso* hypothesis.

### 6.2. CAM Heterodimers

Encouraged by the promising properties of hybrid drugs, the group of Yu designed and synthesized a series of heterodimers comprised of neomycin B, an aminoglycoside antibiotic that binds to the decoding center of the ribosome and CAM [114]. The most potent member of them, NC2 (compound **38**), exhibited one-order higher affinity to model 16S rRNA than that of neomycin B and good discrimination factor between bacterial and human A-sites. However, the antimicrobial activity of NC2 against a panel of pathogenic bacteria was lower than that of neomycin B, probably due to its lower compliance with the Lipinski’s rule of 5 [63].

New CAM heterodimers with improved antimicrobial properties were recently synthesized and evaluated by Berkov-Zrihen *et al.* [115]. We prefer to use the term “heterodimers” for these compounds because they are constructed by connecting two different antibiotics through a linker. The best two of them are presented in Figure 11 (compounds **39** and **40**). Compound **39** was derived from tobramycin, an aminoglycoside that binds to the decoding region of the ribosome and CAM. It exhibited high activity in inhibiting an *in vitro* prokaryotic translating system, better than CAM. It also exhibited good antibacterial activity, comparable to that of CAM and low sensitivity to CAM acetyltranferase. Compound **40** was derived from clindamycin, a semi-synthetic lincosamide that binds to the 50S ribosomal subunit and inhibits early peptide chain elongation by interfering with both A- and P- sites of the catalytic center [116] and CAM. Compared to clindamycin, compound **40** was equally potent as inhibitor of the *in vitro* translation but 3-fold less potent antibacterial compared to CAM. The antimicrobial activity of compound **40** was strain dependent; in the case of *Streptococcus aureus* ATCC51907, *S. aureus* NorA and *Bacillus anthracis,* it was more potent than CAM, whereas in *Bacillus subtilis* 168, *E. coli* MC1061, *Haemophilus influenzae* and *S. pyogenes* it was worse than the parent compound. Compared to CAM, compound **40** was 40% less sensitive to CAM-acetyltranferase but 74% more sensitive to CAM–nitroreductase [115].

### 6.3. CAM Homodimers

**Figure 12:**
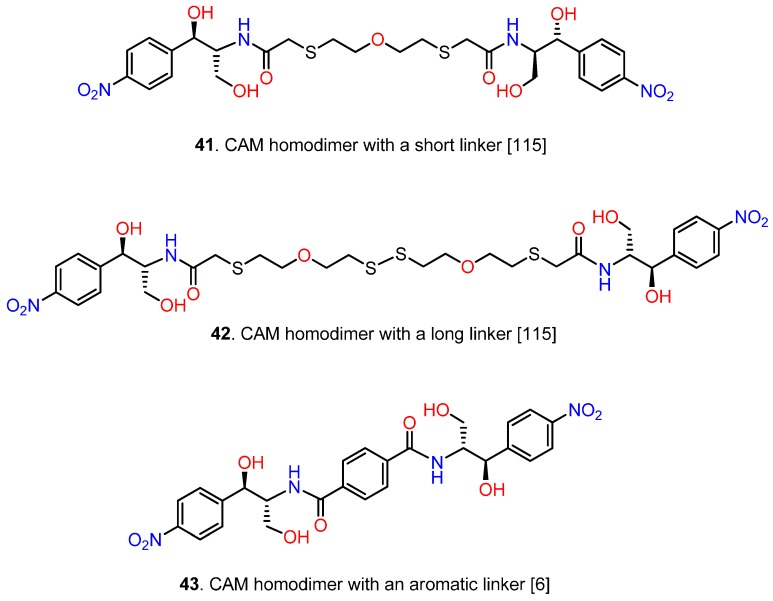
CAM homodimers.

If ribosome possesses two binding sites for an antimicrobial agent and these sites are in close proximity, homodimerization may intensify the action of the agent and improve its potency against resistant strains. This hypothesis was initially examined by Berkov-Zrihen *et al.*, who synthesized homodimers of tobramycin and CAM [115]. CAM homodimers were synthesized by linking bis(2-mercaptoethyl) ether to CAM through a chloroacetyl linker. The intermediate CAM monomer was then transformed to compound **41** (Figure 12).

**Figure 13:**
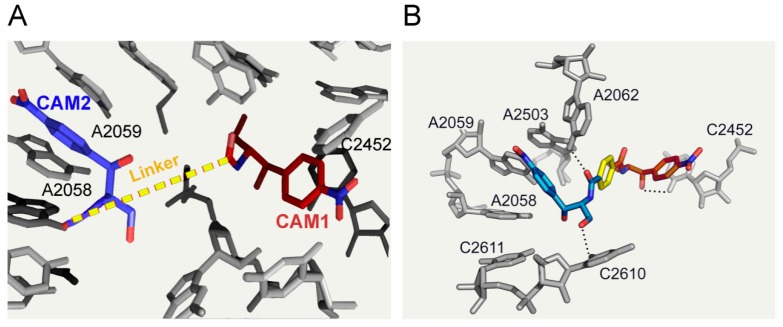
Rationale of CAM dimers designing and binding model of compound **43**. (**A**) Two CAM-binding sites in the *Escherichia coli* ribosome: at the left side, CAM is docked at the entrance to the ribosomal exit tunnel (CAM2) by simulating its crystallographic position in *Haloarcula marismortui* [5]; at the right side, CAM is docked at the A-site of the catalytic crevice (CAM1) by simulating its crystallographic position in *Escherichia coli* [19]. Both molecules are connected by a putative linker and embedded into a nucleotide environment taken from crystallographic data derived from *Escherichia coli*; (**B**) Binding position of compound **43** (in blue, yellow and red) into the *Escherichia coli* 50S ribosomal subunit, as derived by MD simulations [6].

Using a similar strategy another CAM homodimer was prepared, possessing a longer linker (compound **42**). Both compounds, **41** and **42**, demonstrated low inhibitory activities in an *in vitro* prokaryotic translation assay and with a few exceptions, poor antimicrobial activities when compared to CAM. However, homodimerization improved the tolerance against CAM-modifying enzymes. Better homodimers were obtained by our research team in a recent study, using linkers of varying length and flexibility [6]. The rational design of this series of agents was based on the hypothesis that binding of the first CAM unit to the high affinity site (CAM1) could provide the second CAM unit with high local concentration, thus facilitating targeting of the low affinity site (CAM2). Prerequisite for a successful outcome was a correctly adjusted linker, with appropriate length and flexibility. The optimal length was calculated from crystallographic models (Figure 13A) [5,19], while the role of linker flexibility was tested by SAR experiments. Compound **43** incorporates these optimal characteristics; it contains two CAM base units conjugated through a *p*-dicarboxyl aromatic linker of six successive carbon bonds (Figure 12). Compound **43** was found by kinetic, footprinting and cross-linking tests to bind simultaneously within both the entrance to the exit tunnel (nucleotide C2610) and the ribosomal catalytic center (nucleotide C2452) (Figure 13B). Compared to CAM, compound **43** was three-fold more potent in inhibiting the AcPhe-puromycin synthesis. Moreover, it showed similar antibacterial activity against wild-type or MRSA strains of *S. aureus* and wild type Gram-negative bacteria, but superior activity against resistant strains of *Pseudomonas aeruginosa* and *E. coli.* Efforts to improve the cell penetration properties of homodimer **43** are currently in progress.

Compound **43** transiently displayed 5% toxicity on neutrophils during exposure of human blood cells to 60 μΜ of this compound, peaked at 48 h [6]. In comparison, CAM at the same concentration caused 12% toxicity. Toxicity of compound **43** against human monocytes or lymphocytes was negligible. However, compound **43** at 60μΜ induced 43% apoptosis to Jurkat cells, while CAM was almost inactive under the same conditions of treatment. This confers compound **43** a significant advantage over CAM, since the apoptotic behavior of **43** does not allow proliferating cells to continuously survive.

## 7. Synopsis and Future Perspectives

The pharmaceutical behavior of CAM has received dithyrambic praise and severe criticism during its stormy history. Its efficacious activity against a broad spectrum of pathogenic bacteria is hampered by adverse effects causing hematologic disorders, immunosuppression and cancer invasion. As CAM is an ancient microbial metabolite, genetic elements conferring resistance against this drug have been retained by and are frequently dispersed in microbial communities. In parallel, resistance was expanded by the misuse of CAM in the medical and veterinary practice. Currently, CAM is indicated in developed countries only for the treatment of serious infections, in which alternative medication is ineffective or contraindicated. For these reasons, CAM has been modified using various synthetic approaches aiming to optimize it pharmaceutical profile. Despite important progress made to address problems related to side effects and resistance against CAM caused by CAM-modifying enzymes, the Achilles’ heel of the new synthesized CAM derivatives seems to be their general inability to penetrate the bacterial cell envelope, coupled with their susceptibility to multi-drug efflux pumps. Engineering nanolayered particles for specific CAM delivery is a challenge for the future.

It is clear from the past six decades of efforts that CAM derivatives, with ideally improved pharmaceutical properties are incredibly difficult to be found and that there is an urgent need to change our current research ideas in developing new drugs. For example, much of the work toward elucidating the molecular basis of the CAM–ribosome interactions and the mechanisms of resistance has been conducted using model organisms. However, it has been recognized that conclusions emerged from such organisms cannot safely extrapolate to clinical pathogens of interest. Therefore, deeper understanding of the biology and the interactions with the drug of a variety of microorganisms is needed. Similarly, exploring how mitoribosomes interact with a small molecule like CAM and how this drug is metabolized in the host organism, will be important for understanding issues related to drug specificity, resistance and side effects; noteworthy, the mechanisms of the irreversible aplastic anemia following administration of CAM has not been completely established. It should be also realized that while crystallographic studies of CAM complexes with empty ribosomes can provide critical insights into the interactions of CAM with the ribosome in crystalline state, the intrinsic dynamics of the ribosome at physiological translation states could lead to additional or alternative binding modes. Determining such structures will be a challenging topic for the future. Finally, we like to mention that combining CAM and proline-rich antimicrobial peptides into a construct would be an elegant strategy to expand the stability and specificity of interactions between the drug and rRNA, thus leading to new and more effective antibiotics.
